# Patient-centered Bedside Education and Traditional Jewish Law and Ethics

**DOI:** 10.5041/RMMJ.10070

**Published:** 2012-01-31

**Authors:** Yigal Shafran, Joel B. Wolowelsky

**Affiliations:** 1 Program in Science, Halacha and Education, Bar Ilan University, Ramat Gan, Israel, Brooklyn, NY, USA; 2 Department of Jewish Philosophy, Yeshivah of Flatbush, Brooklyn, NY, USA

**Keywords:** Bedside education, medical rounds, medical education, Jewish law and ethics

## Abstract

**Background:**

Bedside rounds have long been a time-honored component of medical education. Recently, there have been various recommendations that residency-training programs further incorporate bedside teaching into clinical curricula.

**Objectives:**

To compare these current attitudes regarding bedside education with the position of traditional Jewish law and ethics.

**Methods:**

Relevant medical journal articles and traditional Jewish sources were reviewed.

**Results:**

Halacha (the corpus of traditional Jewish law and ethics) gives greater focus to a patient-centered rather than student-centered bedside education experience.

**Conclusion:**

Residency training programs should give greater consideration to the importance of a patient-centered bedside education experience.

Bedside rounds have long been a time-honored component of medical education, involving performing activities of clinical care at the patient’s bedside. The patient becomes a “text,” so to speak, used to teach student doctors how to better treat other people in the future.

Gonzalo et al.^1^ report that bedside rounds have been declining these past decades, raising concerns by medical education councils and medical educators about the teaching of clinical skills to trainees, which has prompted recommendations that residency training programs further incorporate bedside teaching into clinical curricula. Peltan and Wright^2^ question the actual extent of the decline but agree that bedside teaching should be promoted for its intrinsic value in medical education. In re-evaluating bedside teaching programs, the demand of the Halacha (the corpus of traditional Jewish law and ethics) on a patient-centered rather than student-centered experience deserves heightened consideration.

Recent rabbinic discussions regarding the halachic attitude towards beside education begin with the exegesis of a biblical text; after describing how a priest diagnoses and ritually treats a particular dermatological affliction, the biblical section closes: “This is the law for all manner of plague of *tsara’at* … to teach when it is unclean and when it is clean; this is the law of *tsara’at*.”^3^ The nineteenth-century rabbinic commentator Rabbi Naftali Zvi Yehuda Berlin^4^ focuses on the words “to teach.” Referencing the twelfth-century Talmudist Rabad of Posquires, he explains that the priest would show the affliction to the student priests in the town to teach them how to identify *tsara’at.* The patient was the “text” for student instruction.

This would seem to be ample endorsement of bedside teaching; however, Berlin goes on to explain why “this is the law of *tsara’at*.” In rabbinic thought, *tsara’at*, infelicitously translated as leprosy, is not a medical disease but rather a physical punishment for violating the laws of appropriate speech by gossiping about others or directly embarrassing them. It is embarrassing for a person to be surrounded by a group of strangers who are closely examining his or her body, he says, and the bedside education of the student priests is tit for tat punishment for the embarrassment the patient had previously caused another. “This is the law of *tsara’at*” and not a general policy of medical education. Following this logic, Rabbi Eliezer Waldenberg, the late halachic ethicist for Jerusalem’s Shaare Zedek Hospital, rules^5^ that, given the potentially embarrassing nature of bedside teaching, one may not allow it without explicit prior permission from the patient. In a contemporary secular context, Aldeen and Gisondi^6^ describe specifically asking permission from the patient before entering the room as proper professional etiquette.

Sensitivity towards the patient’s embarrassment is already recorded in the Talmudic discussion of the mitzvah of *Bikkur Holim* (the religious requirement to visit and care for the sick). The duty falls on everyone, obligating even people of high status to visit those of a lower station. However, the Talmud^7^ prohibits visiting patients suffering from intestinal ailments, because they would be embarrassed by their unhygienic state. Yet this sensitivity is tempered by the medical needs of the patient. The Talmud^8^ records that when one of Rabbi Akiva’s students fell ill, the rabbis did not visit him, presumably because of this prohibition. However, Rabbi Akiva entered and cared for him, at which point the student exclaimed, “My teacher, you have restored me to life!” Rabbi Akiva then proclaimed, “Whoever does not visit the sick is close to shedding blood.” Avoiding embarrassment must not lead to avoiding treatment. But it is the patient’s medical needs that trump the prohibition of embarrassing someone, not the benefit that might accrue to future patients by better educating student doctors.

Waldenberg concedes that bedside rounds contribute to the patient’s well-being. Quoting R. Hanina’s remark that “I have learned much from my teachers, more from my colleagues, and the most from my students,”^9^ he notes that the give and take with the students sharpens the analysis of the attending physician and often raises issues concerning the patient at hand that he or she would not have considered, thereby benefiting the patient. In addition, for example, Aldeen and Gisondi^6^ report studies that show that bedside teaching positively affects the patient–physician relationship and increases patient–physician contact time, which also contributes to improved patient education. These emphasize the value of bedside medical rounds in the treatment of the patient at hand.

Nevertheless, almost half of patients in the study by Lehmann et al.^10^ had recommendations for specific changes in the conduct of bedside rounds that not only point to making the current patient (rather than some future patient) the primary focus of the rounds but reflect halachic values as well: Physicians should ask the patient’s permission to conduct a bedside presentation; they should introduce themselves and be seated during the presentation; they should give greater attention to the patient’s privacy; and they should give the patient the opportunity to say more during the presentations.

Respect for privacy is not only a secular legal right but a basic halachic value that flows from the fact that man was created in God’s image. Explains Rabbi Norman Lamm,^11^ Chancellor of Yeshiva University and head of its affiliated rabbinical school: “As God reveals and conceals, so man discloses and withholds. As concealment is an aspect of divine privacy, so is it the expression of human privacy. … For both God and man, therefore, in that they share the character of personality, there must be a tension and balance between privacy and communication, between concealment and disclosure … [There must be] respect for the inviolability of the personal privacy of the individual, whether oneself or another, which is another way of saying respect for the integrity of the self.”

Sitting rather than standing by the patient is likewise not simply good social etiquette. Barring medical necessity, Halacha requires that when visiting the sick one must not stand lording over the patient, because the *Shikhinah* (that is, God’s very presence) hovers over the patient’s head protecting him or her (Talmud Nedarim 40a). Healing and helping requires a sense of humility.

While contemporary suggestions for improving bedside teaching are fully consistent with these halachic values, one contemporary suggestion would need slight modification. In her “tips for improving bedside teaching,” Ramani^12^ includes a recommendation that “Patients need to be told that the encounter is primarily intended for teaching and that certain theoretical discussions may not be applicable to their illness.” The intention of this recommendation is surely to calm the patients and therefore is an important component of the patient-centered model. However, the *primary* message to the patients should be that while certain theoretical discussions may not be applicable to their illness, the bedside encounter will give a wider group of doctors the opportunity to consider additional therapies, offer the patients an opportunity to ask questions, and at the same time have them help educate a new generation of doctors. Patients should be left with the impression that the *primary* focus is on treating them. Medical rounds should encompass not only bedside education but the patient-centered mitzvah of *Bikkur Holim* as well.
